# A Review of the Abscopal Effect in the Era of Immunotherapy

**DOI:** 10.7759/cureus.29620

**Published:** 2022-09-26

**Authors:** Edward Nabrinsky, Jason Macklis, Jacob Bitran

**Affiliations:** 1 Hematology and Oncology, Advocate Lutheran General Hospital, Park Ridge, USA

**Keywords:** malignancy, radiotherapy, radiation, immunotherapy, effect, abscopal

## Abstract

The abscopal effect is a systemic immune response mediated by the effects of radiation on the immune system. This effect has been observed in a number of cancer types in addition to lung cancer, including but not limited to renal cell carcinoma, hepatocellular carcinoma, lymphoma, and melanoma. The combination of radiation therapy and immune checkpoint inhibition (ICI) acts at several stages of the antitumor response, suggesting a mechanism of synergy between the two modalities. This review focuses on recent advances in the understanding of the effect of radiation and immunotherapy in the context of the abscopal effect.

## Introduction and background

The abscopal effect describes tumor regression outside of the irradiated region. First described in 1953 by Mole [1], it has since been demonstrated in many different cancer types after radiation is directed at the primary tumor. Mediated by a systemic anti-tumor immune response, the effect alludes to the regression of non-irradiated metastatic lesions at sites away from the primary site of irradiation [2]. It is estimated that around half of all cancer patients will receive some type of radiation therapy during the course of their treatment [3]. In non-small cell lung cancer (NSCLC), stereotactic body radiation therapy (SBRT) can result in local control rates of up to 90% at five years in early-stage patients [4]. Systemic reviews of case reports have shown that the abscopal effect following radiotherapy has been observed in a number of cancer types in addition to lung cancer, including but not limited to renal cell carcinoma, hepatocellular carcinoma, lymphoma, and melanoma [5]. Both pre-clinical and clinical studies have supported that regression of tumors outside of the irradiation field is mediated by the effects of radiation on the immune system. With the introduction of immunotherapy, the understanding of immune activation by radiation treatment has further strengthened the role of radiation therapy in systemic disease, as well as demonstrated how the two can work synergistically for tumor burden control. Combinations of radiotherapy appear to have an immunostimulatory effect when radiation fields are optimized to induce immunogenic cell death in tumors.

## Review

Pathophysiology of the abscopal effect

Radiation is a physical agent that works to destroy cancer cells, and ionizing radiation forms electrically charged particles that deposit energy in the tissues it passes through, working to kill cancer cells or cause genetic changes resulting in cancer cell death [3]. Cancer cells are not as efficient as normal cells in repairing damage from radiation and this contributes to differential cancer cell death [6]. External beam radiation, delivered from outside the body by aiming high-energy rays at the location of the tumor, is the most common approach in the clinical setting, whereas modalities such as SBRT can deliver high doses of radiation over a smaller number of treatment fractions to well-defined primary and/or oligometastatic tumors anywhere in the body [3]. Radiotherapy is able to induce an immunosuppressive environment via promoting dysfunctionality of T-cells and recruiting myeloid cells that promote tumor growth [4]; however, it can also generative positive effects by stimulating a systemic immune response to control unirradiated cancer as first demonstrated by Demaria and colleagues in 2004 [7].

After irradiation of tumor cells, tumor antigen release is taken up by antigen-presenting cells, such as dendritic cells, which then migrate to lymph nodes and activate CD8+ T-cells that gain the ability to differentiate into cytotoxic T lymphocytes with the ability to migrate to tumor sites and kill tumor cells [4]. Radiotherapy-induced cell death facilitates neoantigen cross-presentation in dendritic cells and CD8+ T-cell stimulation by activating toll-like receptor 4 (TLR4) and type 1 interferon (IFN) signaling [8]. Immunogenic cell death releases damage-associated molecular patterns (DAMPs), such as high-motility group box 1 (HMGB1), heat shock proteins (HSP), calreticulin membrane exposure, and glucose-regulated protein 96 (GP96), which, in turn, activate dendritic cells through the TLR4 pathway [8]. The HMB1 protein has the ability to stimulate monocytes to produce cytokines such as TNF-a and the interleukins IL-1, IL-6, and IL-8, which act as pro-inflammatory mediators and contribute to dendritic cell maturation [9]. Surviving irradiated cells increase the expression of death receptors, including Fas, and upregulate major histocompatibility class 1 molecules, improving their recognition and killing by activated tumor antigen-specific CD8+ T-cells [10].

The biological target of radiation in the cell is DNA, and through both direct and indirect effects, this cumulates in DNA breaks, with double-strand breaks responsible for most of the tumor cell killing [3]. Double-strand breaks lead to the formation of micronuclei, which ultimately can trigger the stimulation of IFN genes [8]. The STING pathway, activated by IFN genes, is also activated in dendritic cells and promotes anti-tumor CD8+ T-cells in tumor-draining lymph nodes (TLDNs), which play a critical role in T-cell stimulation by concentrating tumor antigens [11]. Buchwald and colleagues demonstrated that TLDN irradiation blunted distant tumor control and that tumor-specific stem-like CD8+ T-cells in TLDN provide a reservoir for terminally differentiated effectors in the tumor, which are capable of tumor cell killing [11]. Additionally, there appears to be a progenitor subpopulation of CD8+ T-cells both within the tumor and TLDNs that are critical for robust PD-1 therapy responses, suggesting a mechanism of synergy between radiation and immunotherapy, which will be discussed later in this review [8].

The tumor microenvironment can be affected by ionizing radiation. Radiation can lead to re-oxygenation of hypoxic tumors, thereby alternating the expression of levels of hypoxia-inducible factor 1a (HIF-1a) and potentially altering the ability of tumors to continue to utilize mechanisms of angiogenesis to further metastasis [1]. Cytosolic double-stranded DNA generated by radiation leads to the production of IFN beta by irradiated cancer cells in addition to the dendritic cancer cells infiltrating the tumor, thereby promoting cross-presentation of tumor antigens to CD8+ T-cells [12]. Surviving cancer cells after radiation exposure become targets for elimination by natural killer and CD8+ T-cells [13]. Another proposed mechanism is the increased surface MHC class I expression and Fas/CD95 interaction that are able to increase rejection of the irradiated tumor by adoptively transferred T-cells, with alterations in the post-radiation transcriptome resulting in changes of the antigenic capabilities by the cancer cells on MHC class 1 [14].

The abscopal effect across tumor types

The abscopal effect has been observed across multiple tumor types. A systematic review from 2016 published by Aboudeh and colleagues reported 46 total cases from 1969-2014 [5]. In this report, non-irradiated, distant responses typically occurred two months after radiation; the median radiation dose was 31 Gy [5]. Case reports with primary tumors, including melanoma, cholangiocarcinoma, and renal cell carcinoma, have described the effect [15-17]. Wersäll and colleagues reported that among 28 renal cell carcinoma patients with treated and untreated metastatic lesions, four patients with non-irradiated metastases had regression either temporarily or permanently after treatment with stereotactic radiation therapy of either the primary tumor or other metastatic lesions; the frequency of such an event was compared to conventional treatments, such as interferon and interleukin-2, and noted to be high compared to rates of spontaneous regression of 0.3-7% in the literature [17]. The authors of this study also noted improved survival time and time to death for the patients who demonstrated the abscopal effect compared to those that did not [18].

Liu and colleagues noted that in case the abscopal effect was observed in intrahepatic cholangiocarcinomas, radiotherapy acted to enhance the presentation of tumor-associated antigens and aid in T-cell recognition [15]. Another study reporting on patients with non-small-cell lung cancer who were not candidates for standard-of-care chemoradiation demonstrated that for patients who were assigned to SBRT to the hypoxic core of primary tumor alone, one-year overall survival was 15% better compared to systemic therapy although the results were not statistically significant [18]. Additionally, reported data on patients receiving risk-adapted targeted intraoperative radiotherapy during lumpectomy for breast cancer suggested that in comparison to patients receiving external beam radiation therapy, patients that received intraoperative radiotherapy with local recurrence did not have worse survival outcomes and therefore may have affected tumor growth at distant sites [19].

Immunotherapy in combination with radiotherapy

The combination of radiation therapy and immune checkpoint inhibition (ICI) acts at several stages of the antitumor response [4]. Radiation boosts ICI by either stimulating exhausted intratumoral CD8+ T-cells or proliferation and differentiation of naive T-cells. Twyman-Saint Victor and colleagues reported the ability of anti-CTLA4 to promote the expansion of T-cells while radiation treatment shaped the T-cell capabilities of the expanded peripheral clones [20]. Additionally, they reported that PD-L1 blockade reversed T-cell exhaustion to mitigate depression in the CD+ T-cell/T regulatory cell ratio and further encourage oligo-clonal T-cell expansion [20]. This reflects the overall findings that CTLA-4 antagonists mainly act on naive, regulatory T-cells while anti-PD1 agents work on newly activated and exhausted T-cells. With these concepts in mind, multiple studies have been done to help determine the most effective sequencing of radiotherapy and ICI. In regards to anti-CTLA-4, treatment in combination with radiation can be effective even before radiation begins due to the depletion of T regulatory cells [21]. A phase I study by Hiniker and colleagues demonstrated that ipilimumab delivered before radiotherapy showed a 50% complete or partial response rate as well as a 27% complete response rate, compared to an 18% partial response rate when it was administered after radiation therapy [22]. Specifically, this study delivered radiation within five days of the first dose of ipilimumab being given [22].

When focusing on the anti-PD1/PDL1 combination with radiotherapy, Dovedi and colleagues suggested that upregulation of anti-PD1 occurs rapidly after radiotherapy and that the mechanism responsible for PD-L1 upregulation in tumor cells involved the production of IFN-y by tumor-infiltrating CD8+ T-cells; therefore, acquired resistance to radiotherapy can be avoided by concurrent administration of PD-L1 with radiotherapy [23,24]. The strategy of administering concomitant ICI with radiation therapy is also supported by a 2014 study reporting that a single dose of radiotherapy increased both tumor cell and monocyte-derived suppressor cell expression of PD-L1 [25]. Dagoglu and colleagues' review reported that in most cases analyzed, the abscopal response occurred in patients who received radiation concurrently with, or immediately following, immunotherapy, with 15/24 studies using ipilimumab, two of 24 using pembrolizumab and three of 24 using nivolumab [26].

Several large, phase 3 prospective randomized clinical trials added immunotherapy after the completion of radiation therapy. The PACIFIC trial showed an overall survival improvement for patients with locally advanced NSCLC treated with adjuvant durvalumab, an anti-PD-L1 antibody, after definitive intent fractionated chemoradiation, which changed the standard of care for these patients [27]. CheckMate 577, a phase 3 prospective randomized trial, showed that the addition of adjuvant nivolumab following chemoradiation and surgery for locally advanced esophageal cancer more than doubled the median disease-free survival [28]. The PEMBRO-RT study, a phase 2 prospective randomized trial where SBRT was administered prior to pembrolizumab, showed a doubling of the response rate in patients with metastatic NSCLC [29], although it was not powered to show a survival benefit. Other strategies that have been studied with radiation therapy include granulocyte-macrophage colony-stimulating factor, intralesional injections, and following circulating tumor DNA [30-32]. Overall, the data support the fact that with the development of ICI, the abscopal effect of radiotherapy has become more frequent.

The abscopal effect with surgical resection

Several reports of the abscopal effect have been described in patients undergoing surgical resection of tumors. Oronsky and colleagues reported three late-stage immunotherapy-treated patients who underwent metastasectomy/debulking and achieved favorable outcomes. In particular, one patient who underwent repair of an abdominal wall defect after previously undergoing debulking surgery of enlarging abdominal masses that were deemed inoperable six months after completing nivolumab and ipilimumab was found to have complete disappearance of the tumor confirmed on positron emission tomography (PET)/computed tomography (CT) [33]. Immunotherapy was continued during the perioperative period in two of the three patients in the guise that it would help drive pro-inflammatory cytokine and chemokine release [33]. Masue and colleagues reported a case of lung metastases in a 58-year-old man with metastatic sarcomatoid carcinoma of the renal pelvis that disappeared spontaneously after nephroureterectomy, with no disease seen 46 months postoperatively [34].

One school of thought regarding this effect is that there are specific molecules or macromolecules that enable the primary tumor to control the growth of metastatic tumors in a particular manner and that surgery may act as a type of trigger on this pathway. Specifically, Seidi and colleagues describe how osteopontin is necessary for bone marrow cell activation and subsequent outgrowth of distant tumors, and that molecules similar to this may be affected by surgery [35]. Further evidence of the abscopal phenomenon in surgical cases was demonstrated by Hamilton and colleagues, who reported a 47-year-old male with brain metastases from NSCLC who went into remission following stereotactic radiosurgery treatment to a brain lesion in the absence of systemic treatment [36].​​​​​​​

Predictors of the abscopal effect

Due to the abscopal effect being rarely observed in literature over time and with increasing awareness of this phenomenon, there has been an effort to understand if any specific factors mediate the effect. Camphausen and colleagues examined whether p53, a protein complex that is up-regulated in irradiated cells, contributed. Overall, they determined that radiotherapy may lead to a systemic antiangiogenic effect mediated through p53 and that this may be one of the mechanisms of the abscopal effect [37]. Specifically, in comparison to mice with wild-type p53 implanted with Lewis lung carcinoma and fibrosarcoma that grew at a slower rate, mice treated with pifithrin-alpha, a p53 blocker, with tumor irradiation did not experience delayed growth [38].

The presence of infiltrating T-cells in certain tumors is associated with improved clinical outcomes, and there has been a significant association between the abscopal effect and the absolute lymphocyte count prior to radiation therapy, making this a potentially useful biomarker for predicting who will exhibit this phenomenon [37]. Other studies have demonstrated that radiation treatment results in increased mutant KPNA2 expression correlating with an upregulation of T-cell receptors, suggesting the possibility of these mutations to predict the response of antitumor T-cells leading to the abscopal effect. T-cell receptor clones reacting to this mutant were almost entirely absent prior to radiation but had dramatic expansion in peripheral blood samples after radiation [39]. Other biomarkers that may mediate the abscopal effect include TREX1, INF-B, as well as phosphorylated histone H2AX [40].​​​​​​​

Future directions

While most of the promising data with combined radiation and immunotherapy have come from patients with melanoma and lung cancer, ongoing studies looking at response rates in other cancer histologies are needed. Additionally, different protocols studying combinations of radiation/immunotherapy are being developed in both limited and extensive-stage lung cancer, non-small cell lung cancer, bladder cancer, and genitourinary malignancies, among others. Janopaul-Naylor and colleague's review analysis highlighted nine ongoing clinical trials evaluating abscopal responses and/or combination radiation and immunotherapy, including in colorectal, non-small cell lung, classical Hodgkin lymphomas, cancer of unknown primary, and mesothelioma [8]. They note that modalities such as ICAM-specific PET imaging may provide future patients with prognostic and predictive data [8]. Craig and colleagues note that biomarkers may be able to be combined with post-treatment indicators to maximize the abscopal effect outcomes of radiation and immunotherapy combinations [40].

## Conclusions

Since its original description in the 1950s, the phenomenon of the abscopal effect has been observed in a variety of tumor types and settings. Whereas historically, the effect was rarely observed and limited to an association with radiation, the recent advent and expansion of immunotherapy have added to a new realm in the observation and benefit of the abscopal effect. Additionally, the recognition of several different biomarkers has helped shape our understanding of which patients are more likely to have the abscopal effect. Different combination strategies have led to beneficial responses for patients, and new strategies in the context of clinical trials are continuing to be developed. We look forward to the continued development and understanding of strategies that will benefit patients across a variety of tumor types.
